# High levels of dietary soy decrease mammary tumor latency and increase incidence in MTB-IGFIR transgenic mice

**DOI:** 10.1186/s12885-015-1037-z

**Published:** 2015-02-06

**Authors:** Katrina L Watson, Leanne Stalker, Robert A Jones, Roger A Moorehead

**Affiliations:** Department of Biomedical Sciences, Ontario Veterinary College, University of Guelph, 50 Stone Road East, Guelph, ON N1G2W1 Canada

**Keywords:** Breast cancer, Soy, IGF-IR, Tumor initiation, Tumor progression, Metastasis, Metaplastic breast cancer, Amphiregulin

## Abstract

**Background:**

Epidemiologic data indicates that Asian diets, which are high in soy protein, reduce a women’s risk of developing breast cancer. However, it has been difficult to dissociate the benefits of soy from other variables including environmental and lifestyle factors. Since prospective studies in humans would take decades to complete, rodent models provide a valuable research alternative.

**Methods:**

In this study, MTB-IGFIR transgenic mice, which develop mammary tumors resulting from overexpression of the type I insulin-like growth factor receptor (IGF-IR), were utilized. MTB-IGFIR mice were fed a soy-based or casein-based diet throughout all stages of development to reflect soy exposure in Asian cultures. Mammary tumors were initiated at 2 different developmental stages by commencing IGF-IR transgene expression either during puberty or in adult mice.

**Results:**

MTB-IGFIR mice fed a soy-based diet displayed increased tumor incidence and accelerated tumor onset compared to MTB-IGFIR mice fed a casein diet. Two markers of estrogen receptor signaling, *Pgr* and *Areg*, were elevated in mammary tissue from mice fed the soy diet compared to mice fed the casein diet suggesting that high levels of soy may promote mammary tumor development through acting as an estrogen receptor agonist. Mammary tumors from mice fed a soy diet more frequently expressed metaplastic markers such as cytokeratins 5 and 14 as well as p63 and displayed reduced lung metastases compared to mammary tumors from mice fed a casein diet.

**Conclusions:**

Diets consisting of very high levels of soy protein promote mammary tumor development and decrease tumor latency possibly through activating estrogen receptor signaling. Additional studies are required to determine whether a more moderate amount of dietary soy can inhibit oncogene-induced mammary tumorigenesis.

**Electronic supplementary material:**

The online version of this article (doi:10.1186/s12885-015-1037-z) contains supplementary material, which is available to authorized users.

## Background

Breast cancer rates vary depending on geographical location with the incidence of breast cancer being relatively high in North America and Western Europe compared to Asian countries such as Japan and China [1]. One factor implicated in contributing to the lower breast cancer rates observed in Asian countries is the high consumption of soy products. In countries such as China, soy consumption ranges from 27-141 g of soy per day while individuals born in the United States, Canada and Western Europe typically consume less than 4 g of soy per day [2].

Soy has been shown to contain several putative chemopreventative agents including isoflavones [3]. Isoflavones are plant derived phytoestrogens and the main isoflavones found in soy are genistein, daidzein and glycitein [4]. Isoflavones bind weakly to estrogen receptors (ERα and ERβ) and appear to preferentially bind to ERβ [5-8]. Based on their affinity for ERs it has been proposed that soy isoflavones can reduce breast cancer risk by interfering with the binding of endogenous estrogens to ERs thus suppressing ER signaling.

The protective effects of soy have been demonstrated in case–control and cohort studies in Asian populations in that soy consumption was inversely associated with breast cancer risk [9-13]. Reductions in risk of 29% have been reported when women consuming high levels of soy isoflavones (>20 mg/day) are compared to those consuming low levels of soy isoflavones (<5 mg/day) [14]. However, a subset of studies failed to show a benefit in soy consumption with respect to breast cancer. Three case–control, six cohort and six prospective studies have failed to demonstrate any significant association between soy consumption and breast cancer risk (reviewed in [4]). One of these studies actually showed that soy consumption was associated with increased breast cancer risk [15]. A very recent study by Shike et al. [16] demonstrated in a randomized-placebo controlled study that soy supplementation for 7–30 days in women with early stage breast cancer increased the expression of a number of cell cycle related genes compared to women receiving a placebo. Therefore, under certain conditions soy consumption may provide little or no benefit and in some scenarios, have detrimental effects.

Since it is difficult to design studies to assess the causal association between dietary soy and mammary tumor risk as well as the mechanism through which soy modifies mammary tumorigenesis in humans, animal models have also been employed. Most of the animal studies performed to date have explored the impact of soy or specific isoflavones on chemically induced mammary tumors in rodents. Using these models, it has been shown that soy or specific isoflavones inhibit mammary tumor development induced by chemicals such as 7,12-dimethylbenz(a)anthracene (DMBA) and nitrosomethylurea (NMU) [17-22]. It should be noted that a small number of studies have failed to demonstrate a protective effect of soy isoflavones and in some situations dietary soy consumption enhanced chemically-induced mammary tumorigenesis [23-25].

More recently, transgenic mice have been used to determine whether dietary soy protects against mammary tumor development. MMTV-Wnt1 mice fed a soy diet after weaning exhibited reduced mammary tumor incidence but significantly shorter tumor latency compared to mice fed a casein diet [26]. In a study using MMTV-erbB2 transgenic mice, the animals were manipulated to have low, moderate or high levels of estrogen and then placed on a soy or control diet 2 weeks after hormone manipulation. This study found that soy significantly induced mammary tumor development in the low estrogen group, inhibited mammary tumor development in the high estrogen group and had no effect on the moderate estrogen group [27]. Another study using MMTV-erbB2 transgenics showed that initiating a soy diet at 2 months of age had no effect on mammary tumor development compared to a soy-free diet [28].

The animal studies performed to date do suffer from some limitations. First, diets high in soy or isoflavones are often initiated during postnatal development while Asian populations presumably consume high levels of soy throughout their lives and thus Asian women are exposed to soy isoflavones during embryonic and postnatal development. Data from human studies suggest that although soy intake at any age decreases breast cancer risk, the greatest decrease in risk was associated with high dietary soy intake during childhood [12,29-31]. A second limitation is the timing of mammary tumor transformation. DMAB or NMU are often administered as a single, high dose during pubertal development, an induction scenario that is unlikely to recapitulate breast cancer initiating events in humans. The mouse mammary tumor virus (MMTV) promoter which is typically used to drive the expression of transgenes in mouse mammary tissue is active at all stages of development and thus the tumor inducing oncogene is expressed during the later stages of embryonic development and all stages of postnatal development. This pattern of oncogene expression may reflect inherited gene alterations/mutations, however, only 20-25% of breast cancers are thought to be familial [32].

Our study was designed to minimize the limitations of other animal models. To accomplish this, soy or casein diets were administered to female mice prior to mating and the females were maintained on the same soy or casein diet during lactation. Upon weaning the offspring were also maintained on the soy or casein diet to ensure female MTB-IGFIR transgenic mice were exposed to the soy or casein diet throughout all stages of development. Since it remains unclear when breast cancer initiation occurs, the IGF-IR transgene was induced at two different developmental stages, during puberty and in young, post-pubertal mice. Induction of the IGF-IR transgene at these two stages would simulate a spontaneous genetic alteration acquired in pubertal or young adult women.

Using this experimental design, mice fed a soy-based diet had a significant increase in tumor incidence and shorter tumor latency than mice fed a casein-based diet. Mammary tumors from mice fed a soy diet more frequently expressed markers associated with metaplastic breast cancer including cytokeratin 5, cytokeratin 14, p63 and the presence of osseous matrix. The soy diet appeared to have a protective effect against tumor progression as lung metastases were more frequent in casein-fed mice. Although it is unclear how dietary soy promotes mammary tumorigenesis, two estrogen regulated genes, *Pgr* and *Areg* were expressed at higher levels in mammary glands of soy-fed mice suggesting a stimulatory effect of soy on estrogen receptor signaling.

## Methods

### Ethics

Animals were housed and cared for following guidelines established by the Central Animal Facility at the University of Guelph and the guidelines established by the Canadian Council of Animal Care. This study was approved by the Animal Care Committee at the University of Guelph (AUP# 1695) and all efforts were made to minimize suffering.

### Mice

MTB-IGFIR transgenic mice were generated in our lab and have been previously described [16]. At the time of mating, mice were placed on a diet where the sole source of protein was from casein or isolated soy protein and mice were maintained on this diet throughout pregnancy and lactation. Following weaning (21 days of age) the mice were maintained on either the casein or soy diets. At either 45 days of age or 100 days of age female MTB-IGFIR mice were switched to either the casein diet + 100 mg of doxycycline per kilogram of food or the soy diet + 100 mg of doxycycline per kilogram of food. Mice were maintained on the doxycycline supplemented diet until the end of the study. All diets were obtained from Harlan Laboratories (Madison, WI) and the detailed composition of each diet is provided as supplementary data (Additional file 1: Table S1). The isolated soy protein used by Harlan typically contains approximately 463 ppm, 95 ppm, 933 ppm, 101 ppm, 57 ppm and 9 ppm of daidzin, daidzein, genistin, genistein, glycetin and glycetein, respectively with a total isoflavone content of approximately 1660 ppm, all aglycone.

### Tumor onset and growth rate

Mammary tumor onset was determined by palpation. Once a mammary tumor was identified it was measure approximately three times per week using digital calipers. Tumor volume was estimated using the formula: *volume = length x width*^*2*^*/2*. Tumor specific growth rate (SGR) was estimated using the formula: *SGR = ln(V*_*2*_*/V*_*1*_*)/(t*_*2*_*-t*_*1*_*)* [31].

### Histology and immunohistochemistry

Histology and immunohistochemistry were performed as previously described [33]. The antibody against IGF-IR was obtained from R&D Systems (Minneapolis, MN), while antibodies against cytokeratin 5, cytokeratin 8, cytokeratin 14, and p63 were all obtained from Abcam (Toronto, ON, Canada). The p63 antibody was used at a dilution of 1:750 while the antibodies for IGF-IR cytokeratin 5, cytokeratin 8, and cytokeratin 14 were used at a dilution of 1:100.

### Goldner’s trichrome staining

Tissue sections were de-waxed in xylene and then re-hydrated in decreasing concentrations of alcohol ending with water. The sections were then stained for 20 minutes with Weigert’s iron hematoxylin and rinsed in water for 1 minute. Sections were then differentiated by incubating in 1% hydrochloric acid in 70% ethanol for 5 seconds and then rinsed in water. Next, sections were stained for 5 minutes in solution A (0.075% Ponceau 2R, 0.025% acid fuchsin, 0.01% azophloxine and 0.2% acetic acid in distilled water), 3 minutes in solution B (2% orange G and 4% phosphomolybdic acid in distilled water) and 5 minutes in solution C (0.2% light green and 0.2% acetic acid in distilled water). Between solution A and B as well as between solution B and C, the sections were rinsed with 1% acetic acid. Sections were then incubated in 1% acetic acid for 5 minutes, rinsed in 100% alcohol, and mounted. All steps were performed at room temperature.

### Western blotting

Western blotting was performed as previously described [34]. Primary antibodies for Akt, phosphorylated Akt (Ser473), Erk1/2, phosphorylated Erk1/2 (Thr202/Tyr204), STAT3 and phosphorylated STAT3 (Tyr705) were purchased from Cell Signaling Technologies (Beverly, MA) and were used at a 1:1,000 dilution. The primary antibody for IGF-IR was obtained from R&D Systems (Minneapolis, MN) and was used at a 1:2,000 dilution. HRP-conjugated secondary antibodies were obtained from Cell Signaling Technologies (Beverly, MA) and were used at a 1:2,000 dilution. Images were captured on ChemiDoc XRS+ imaging system (Bio-Rad Laboratories, Mississauga, ON Canada) and band intensities were determined using Image Lab software (Bio-Rad Laboratories, Mississauga, ON Canada).

### RNA extraction and real-time PCR

RNA was extracted from mammary tissue using the mirVana miRNA isolation kit (Life Technologies, Burlington, ON, Canada) following the manufacturer’s instructions. RNA was reverse transcribed as previously described [35]. Quantitative RT-PCR was performed using Platinum SYBR Green qPCR SuperMix (Life Technologies, Burlington, ON, Canada) and Cq values were determined using the PrimePCR program on a CFX96 real-time PCR machine (Bio-Rad Laboratories, Mississauga, ON, Canada) as determined by the CFX Manager software v3.1 (Bio-Rad Laboratories, Mississauga, ON, Canada). Primers for *Esr1* and *Pgr* were obtained from Origene (Rockville, MD) while primers for *Areg*, *Fgf10*, *Fgfr1*, *Fgfr2*, *Tgfα*, *Hprt* and *Ywhaz* were obtained from Bio-Rad Laboratories (Mississauga, ON Canada). Primer efficiencies were *Esr1* – 91.3%, *Pgr* – 92.5%, *Areg* – 101.1%, *Fgf10* – 109.3%, *Fgfr1* – 103.2%, *Fgfr2* – 98.6%, *Hprt* – 105.0%, *Tgfα*- 92.1% and *Ywhaz* – 110.0%. Relative gene expression was calculated using CFX manager software version 3.1 (Bio-Rad Laboratories, Mississauga, ON Canada) and *Hprt* and *Ywhaz* were used for normalization. *Hprt* and *Ywhaz* had previously been identified as the as appropriate housekeeping genes from a reference gene panel of 14 genes which also included *Tbp*, *Actb*, *Gusb*, *Gapdh*, *B2m*, *Rps18*, *G6pdx*, *Hmbs*, *Nono*, *Ppia*, *Tfrc* and *Rpl13a* (Bio-Rad Laboratories, Mississauga, ON Canada) using geNorm [36].

### Statistics

Kaplan-Meier curves were generated in SPSS version 20 (IBM, Armonk, NY) and used to compare the tumor onset between the soy and casein diet groups. A Log Rank (Mantel-Cox) test was used to determine whether the Kaplan-Meier curves were significantly different. An ANOVA followed by a Tukey’s post-hoc test was used to compare tumor onset following administration of different concentrations of doxycycline. A Fisher’s Exact test was used to compare the number of mice that developed mammary tumors and the number of tumor bearing mice that developed lung metastases between the two diets. A Student’s T-test was used when comparing gene expression between the two diets. In all cases, p < 0.05 was considered statistically significant.

## Results

### Titration of IGF-IR transgene expression modulates mammary tumor onset in MTB-IGFIR mice

Our original study with the MTB-IGFIR transgenic mice used 2g of doxycycline (DOXC) per litre of water or per kilogram of rodent chow to induce high levels of the IGF-IR transgene in mammary epithelial cells [34,37]. This level of IGF-IR expression induced mammary tumors in the MTB-IGFIR transgenic mice within approximately 50 days [34]. Since mammary tumor onset was so rapid at this level of IGF-IR expression, it may be difficult for dietary alterations to influence mammary tumorigenesis. To establish a more reasonable rate of tumor onset, MTB-IGFIR mice were fed 1 g/kg, 500 mg/kg, 250 mg/kg and 100 mg/kg of DOXC in the animals’ food. It was found that tumor onset was delayed only in the MTB-IGFIR mice fed 100 mg/kg DOXC and using this concentration of DOXC, mammary tumors were induced within average onset of 157 ± 26.5 days (Additional file 2: Figure S1A). The delay in mammary tumor onset was likely due to the significant reduction in IGF-IR protein induced by 100 mg/kg DOXC compared to 2 g/kg DOXC (Additional file 2: Figure S1B,C). 100 mg/kg DOXC was used to induce mammary tumors in this study.

### Tumor onset, incidence, multiplicity and growth rate

To evaluate the effect of dietary soy on oncogene-induced mammary tumorigenesis, mice were maintained on soy-based or casein-based diets throughout embryonic and postnatal development. To achieve this, female mice were exposed to either the soy or casein diet at the time of mating and remained on this diet until the mice were weaned. Once the mice were weaned, they remained on the soy- or casein-based diet until they were euthanized.

The IGF-IR transgene was induced at two different time points, postnatal day 45 (PND45) and PND100. These time points were selected to initiate mammary tumor development in pubertal mice (PND45) or young adult mice (PND100) [38,39]. Mice were monitored for a maximum of one year after the induction of the IGF-IR transgene. Mice that did not develop tumors by this point were euthanized and mammary glands collected.

**Figure 1:**
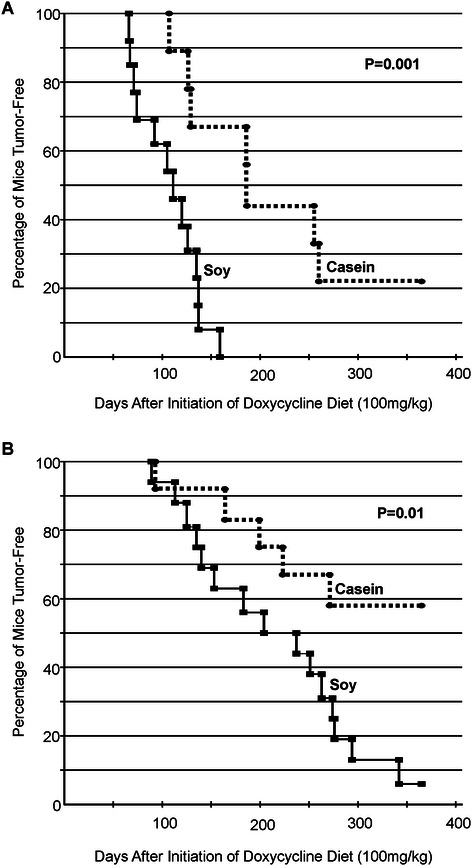
**Kaplan-Meier curves of tumor-free mice fed either a soy (solid line) or casein (dashed line) diet and had the IGF-IR transgene induced at (A) PND45 or (B) PND100.** Tumor development was significantly faster in the soy-fed mice compared to the casein-fed mice at both induction times.

When the IGF-IR transgene was induced at PND45, it was observed that 13/13 of the mice fed the soy diet developed mammary tumors within the first year after IGF-IR transgene induction while only 7/9 mice fed the casein diet developed mammary tumors within this time frame (Table 1). The two casein mice that did not develop mammary tumors had normal mammary glands based on histological analysis and expressed high levels of IGF-IR in mammary epithelial cells (Additional file 3: Figure S2) indicating that the IGF-IR transgene was still expressed at high levels in these mammary glands. Kaplan-Meier curves for tumor onset following IGF-IR induction at PND45 are presented in Figure 1A. Based on these curves it was determined that mice fed the soy diet had significantly faster tumor onset than mice fed the casein diet. Average tumor onset for the mice receiving the soy diet was 108 ± 9 days after IGF-IR transgene induction while average tumor onset for the mice receiving the casein diet was 179 ± 23 days.

**Table 1:** **Mammary tumor development and metastatic spread**

Diet	# of mice	# of mice that developed mammary tumors	Days for mammary tumor to become palpable^1^	Average # of tumors/mouse	Tumor specific growth rate	# of mice with lung metastases
Soy (PND45)	13	13	108 ± 9	2.1 ± 0.3^1^	3.1 ± 0.3	5
Casein (PND45)	9	7	179 ± 23^1^*	1.9 ± 0.4^1^	3.2 ± 0.6	3
Soy (PND100)	16	15	205 ± 20^1^	1.9 ± 0.3^1^	3.2 ± 0.4	4
Casein (PND100)	12	5^**^	190 ± 30^1^	1.6 ± 0.4^1^	3.7 ± 0.3	4

^1^only mice that developed tumors were included.

*p < 0.05 compared to Soy PND45 (Student’s T-test).

**p < 0.05 compared to the number of tumors in Soy PND100 (Fisher’s Exact Test).

When the IGF-IR transgene was induced at PND100, it was observed that 15/16 of the mice fed the soy diet developed mammary tumors within the first year after IGF-IR transgene induction while only 5/12 mice fed the casein diet developed mammary tumors within this time frame (Table 1, Figure 1B). Mammary glands from mice that did not develop mammary tumors, appeared phenotypically normal and expressed high levels of the IGF-IR transgene (data not shown). Kaplan-Meier curves revealed that tumor onset was significantly accelerated in the soy-fed mice compared to the casein-fed mice (Figure 1B).

There was no significant difference in the average number of mammary tumors that developed in each mouse on the two different diets at either time point (Table 1). In addition, tumor specific growth rate (SGR) was not significantly different between the two diets at either time point (Table 1).

### Tumor histology

**Figure 2:**
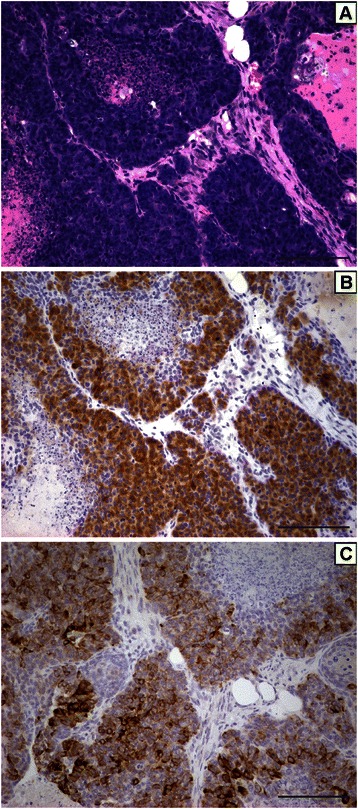
**Representative H&E stained section (A), IGF-IR immunohistochemistry (B) and cytokeratin 8 immunohistochemistry (C) of the mammary tumors that developed in the soy-fed and casein-fed mice following IGF-IR transgene induction.** Scale bars, 100 μM.

Histological evaluation of the mammary tumors revealed that tumor cells from both the casein- and soy-fed mice typically possessed a shape consistent with luminal tumor cells (Figure 2A). IGF-IR immunohistochemistry indicated that most of the tumor cells in both diets expressed moderate to high levels of IGF-IR (Figure 2B). Regions of necrosis were frequently observed in tumors from both diets (Figure 2A). Tumor cells with a luminal morphology typically stained positive for cytokeratin 8 (Figure 2C).

**Figure 3:**
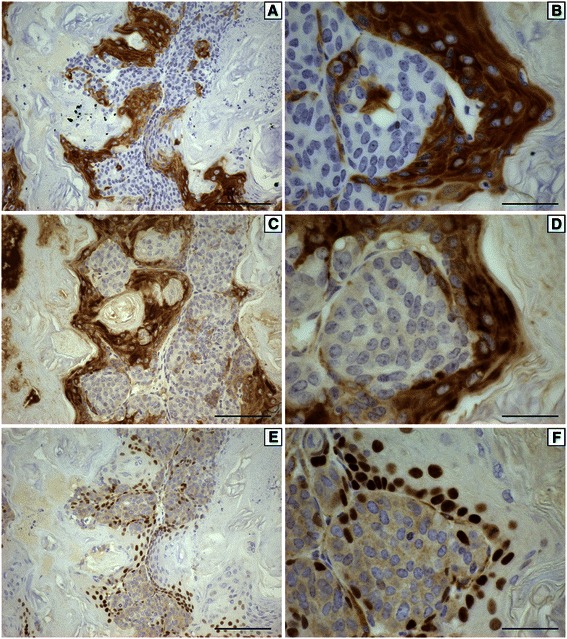
**Representative immunohistochemistry for cytokeratin 5 (A,B), cytokeratin 14 (C,D) and p63 (E,F) from a mammary tumor that developed in a soy-fed mouse following IGF-IR induction at 100 days of age at 200x (A,C,E) or 600x (B,D,F) magnification.** Scale bars in **A,C,E** are 100 μM while scale bars in **B,D,F** are 33 μM.

Another frequent characteristic of these tumors was pockets of cells that stained positive for basal cytokeratins such as cytokeratin 5 (Figure 3A,B) or cytokeratin 14 (Figure 3C,D). Cells staining positive for basal cytokeratins were also frequently positive for p63 (Figure 3E,F). Additional file 4: Figure S3 shows the expression of cytokeratin 5 (CK5), cytokeratin 14 (CK14) or p63 in the different tumors.

**Figure 4:**
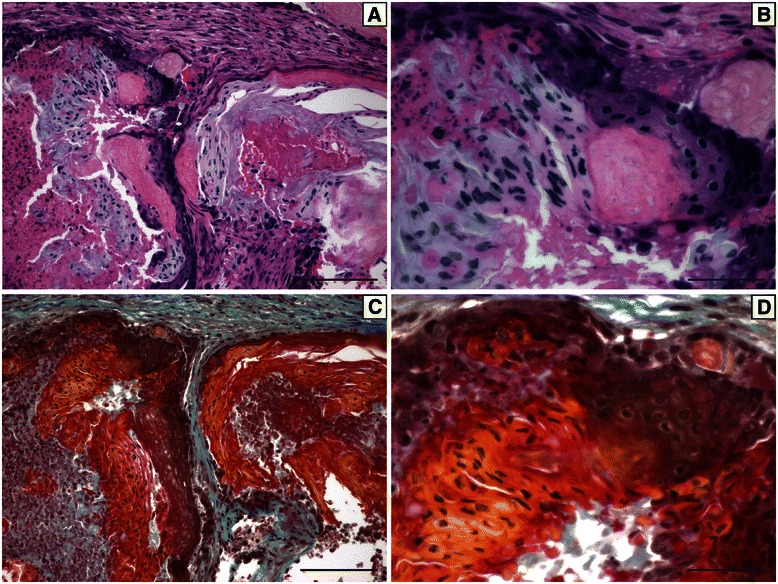
**A representative H&E stained section (A,B) or Goldner’s stained section (C,D) of a mammary tumor from a soy-fed mice with IGF-IR induction at 100 days of age.** A white matrix-like material was observed in five tumors from soy-fed mice and this matrix stained red/orange following Goldner’s staining suggesting the matrix may be osteoid. Scale bars for **A**,**C** are 100 μM while scale bars for **B**,**D** are 33 μM.

Interestingly, a subset of tumors contained a white/grey substance that was visible following H&E staining (Figure 4A,B). This material was only observed in tumors from soy-fed mice when the IGF-IR transgene was induced at 100 days of age (5/15 mice) and was not found in any other tumors. Goldner’s Trichrome staining suggested that these deposits may be a type of matrix such as osteoid (Figure 4C,D).

### Lung metastasis

Lung metastases were identified using IGF-IR immunohistochemistry (Additional file 5: Figure S4A) since mammary tumors that metastasize to the lung maintain high levels of IGF-IR transgene. This approach also allowed for the identification of small lung metastases (Additional file 5: Figure S4B). All lobes of the lungs from each mouse were evaluated in one randomly chosen tissue section.

It was observed that 5/13 (38%) of the soy-fed mice that developed mammary tumors had at least one metastatic lesion while 3/7 (43%) of the casein-fed mice that developed mammary tumors had at least one metastatic lesion when the IGF-IR transgene was induced at PND45 (Table 1). When the IGF-IR transgene was induced at PND100, it was observed that 4/15 (27%) of the soy-fed mice had at least one metastatic lesion while 4/5 (80%) of the casein-fed mice had at least one metastatic lesion (Table 1). The metastatic frequency of the casein-fed mice was nearly statistically different from the soy-fed mice when the IGF-IR transgene was induced at PND100 (p = 0.054). When the data are pooled, 7/12 (58%) casein-fed and 9/28 (32%) soy-fed mice developed lung metastases (p = 0.087).

When the lungs were examined from tumor bearing mice individual lobes would typically contain 0–4 lung metastases and the total number of lung metastases in a tumor-bearing mouse ranged from 1–8 metastases. However, one mouse fed the casein diet and IGF-IR transgene induction at PND45 developed multiple lung metastases throughout each lobe (Additional file 6: Figure S5A,B). It remains unclear why this one mouse had such extensive metastatic burden. Analysis of the primary mammary tumor revealed that the tumor was composed of cells with luminal appearance that maintained some glandular structure and had considerable amounts of intervening stroma (Additional file 6: Figure S5C). Only 1 other mouse had a mammary tumor with similar morphologic features and this mouse was fed the casein diet and had the IGF-IR transgene induced at PND100 (data not shown). While this tumor metastasized to the lung only 3 small lung metastases were visible across all the lung lobes (data not shown).

There was no consistent difference with respect to the total number of lung metastases or the size of the lesions between the two diets at either time point of IGF-IR induction.

### IGF-IR expression and downstream signaling

**Figure 5:**
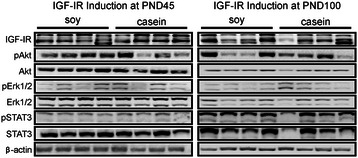
**Western blot analysis for IGF-IR, Akt, phosphorylated Akt (Ser473), Erk1/2, phosphorylated Erk1/2 (Thr202/Tyr204), STAT3 and phosphorylated STAT3 (Tyr705) in mammary tumors from mice fed soy or casein diets.** β-actin served as a loading control.

Western blotting was performed on tumors taken from four independent mice for each diet when the IGF-IR was induced at PND45 or PND100. Tumors were evaluated for IGF-IR, Akt, Erk1/2 and STAT3 as well as the phosphorylated forms of Akt, Erk1/2 and STAT3. As shown in Figure 5 there were no obvious differences in the levels of IGF-IR, Akt, Erk1/2 or STAT3 in the mammary tumors derived from mice fed soy or casein diets.

### Gene expression in Soy and casein mammary glands

Phytoestrogens in soy are capable of activating estrogen receptors albeit with a lower potency than endogenous estrogens. To evaluate whether a diet where soy was the sole source of protein could influence estrogen receptor level or signalling, RNA was extracted from mammary glands of 45 day old, MTB-IGFIR mice fed a soy or casein diet throughout embryonic and postnatal development that were not exposed to DOXC. As shown in Table 2, the soy diet did not significantly change the expression levels of estrogen receptor alpha (*Esr1*). Progesterone receptor (*Pgr*) expression was approximately 2.2-fold higher in the soy-fed mice compared to the casein-fed mice but this difference was not statistically significant as there was a considerable amount of variation in the different mammary samples.

**Table 2:** **Gene expression in mammary glands from mice fed soy or casein diets**

Gene	Soy mean ± SEM^1^	Casein mean ± SEM^1^	Fold difference (soy relative to casein)
*Esr1*	2.56 ± 0.37	3.64 ± 0.88	0.7
*Pgr*	0.24 ± 0.08	0.11 ± 0.04	2.2
*Areg*	1.43 ± 0.32	0.57 ± 0.18*	2.5
*Tgfα*	0.13 ± 0.03	0.12 ± 0.03	1.2
*Fgf10*	1.32 ± 0.09	2.10 ± 0.41	0.6
*Fgfr1*	3.65 ± 0.58	4.80 ± 0.92	0.8
*Fgfr2*	1.01 ± 0.07	0.79 ± 0.20	1.3

^1^normalized to *Hprt* and *Ywhaz.*

*p < 0.05, Student’s t-test.

The expression of additional regulators of mammary epithelial proliferation were also investigated including amphiregulin (*Areg*), fibroblast growth factor 10 (*Fgf10*), FGF receptor 1 (*Fgfr1*), *Fgfr2*, and transforming growth factor alpha (*Tgfα*). As shown in Table 2, high levels of *Fgf10* and *Fgfr1*, moderate levels of *Fgfr2* and *Areg* and only low levels of *Tgfα* were found in the mammary tissue. The only gene that was significantly elevated in the mammary glands of soy-fed mice was *Areg* and this gene was expressed approximately 2.5-fold higher in mammary glands from soy fed compared to mammary glands from casein fed mice.

## Discussion

The goal of this project was to determine whether a diet where soy was the sole source of protein could protect against the development of mammary tumors mediated by the mammary-tumor inducing oncogene, IGF-IR. To model soy exposure in Asian cultures, mice were exposed to soy protein throughout embryonic development and during nursing by feeding the mothers of the offspring diets high in soy or diets lacking soy and the mice were maintained on the high soy or soy-free diet upon weaning. Since there is still considerable debate regarding the age when the initial events of mammary tumorigenesis occur, mammary tumors were induced at two time points; in pubertal mice and in young adult mice.

In our study, animals fed the high soy diet displayed increased mammary tumor incidence and shorter tumor latency compared to mice fed a soy-free, casein-based diet. This enhanced mammary tumor incidence and onset was observed when the IGF-IR transgene was induced in mammary epithelial cells at 45 and 100 days of age. This finding is surprising considering that studies in humans suggest that a diet high in soy protein protects against mammary tumor development. The main difference between our study and those carried out in humans is that in our work 100% of the dietary protein came from soy protein. Asian cultures consume ~20-141 g of soy per day [2]. Using 55 kg as an estimate of the average weight for an Asian women, the upper limit of soy consumption would be approximately 2.6 g of soy/kg body weight/day. Mice eat approximately 5 g/day and the diet provided to our mice contained 0.2 g of soy protein/g of food. With an average weight of 25 g, our mice were consumed approximately 40 g of soy/kg body weight/day. This soy consumption is ~15-fold higher than the upper amount of soy reported to be consumed by Asian women. Therefore, it is possible that very high levels of soy used in our study acted as a tumor promoter, whereas more moderate soy intake may prevent mammary tumorigenesis.

A majority of the animal studies evaluating the chemopreventative effects of soy have been performed in rats exposed to chemical carcinogens. These studies typically do not initiate soy administration until the postnatal period and thus it is difficult to compare our findings to these studies. Two studies did however evaluate the impact of dietary soy on mammary tumor development when rats were exposed to soy during embryogenesis. In a study by Hilaviki-Clarke et al., daily subcutaneous injections of 20, 100 or 300 ug of genistein during days 15 and 20 of gestation, increased the incidence of mammary cancer following DMBA administration to rats at 2 months of age [40]. Lamartiniere et al., fed rats diets containing 0, 25 or 250 mg of genistein/kg of food beginning two weeks before mating and the offspring were maintained on the diet until weaning at which point the female offspring were placed on a soy-free diet. DMBA was administered at 50 days of age to initiate tumor development. Using this design, both the 25 and 250 mg of genistein inhibited mammary tumor multiplicity [41]. Lamartiniere et al. then altered the soy exposure such that females were fed a diet containing 250 mg of genistein/kg of food only during breeding and pregnancy and females were switched to a soy-free diet at parturition. Feeding soy only during breeding and pregnancy did not significantly reduce the tumor multiplicity compared to control mice [41] suggesting that soy exposure during the embryonic phase had no effect on mammary tumor development in this study.

Evaluation of normal mammary development also suggests that soy isoflavones can result in changes in the mammary gland that may promote tumor development. In one study, administration of the soy metabolite, equol, to rats during neonatal development led to precocious mammary gland differentiation compared to prepubertal treatment (days 21–35) [30]. In addition, rats administered genistein during embryonic development and lactation had abnormal mammary gland histology including regions of hyperplasia and increased mammary epithelial cell proliferation in both ducts and terminal end buds [42]. Finally, administration of increasing concentrations of genistein during embryonic and postnatal development induced mammary gland hyperplasia in both male and female rats at PND50 when genistein was administered at 250 ppm or higher [43].

Studies administering soy diets to transgenic mice have also shown conflicting results. A study by Chiesa et al. [44] fed MMTV-neu transgenic mice a diet high in soy protein and soy isoflavones during gestation and lactation. At weaning, mice received a soy-free diet, a diet high in soy protein and soy isoflavones or a diet high in soy protein but containing low levels of soy isoflavones. All mice developed mammary tumors and the mice fed an isoflavone-poor, soy protein concentrate had significantly lower tumor weight at the time of sacrifice (5 months of age). In a study by Luijten et al. [45] diets containing low, moderate or high levels of soy isoflavones were administered either during pregnancy and lactation or at 4 weeks of age to MMTV-neu transgenic mice. In the perinatal exposure group, the average number of tumors and total tumor mass was significantly higher in the medium and high soy diet groups. In the post-weaning study, soy had no effect on tumor incidence, tumor multiplicity or total tumor mass. Two studies that evaluated the impact of soy administration to pubertal MMTV-neu transgenic mice found that soy administration delayed mammary tumor development [46,47]. Thus, the impact of dietary soy on oncogene-induced mammary tumor development requires further investigation to determine whether soy consumption can prevent mammary tumor development and if so, the optimal amount of soy that should be consumed and the developmental window that soy consumption would provide the greatest benefit.

The mechanism through which soy isoflavones regulate mammary tumorigenesis remains unclear but most likely involves signaling through ERs. Estrogen is one of the main regulators of mammary epithelial proliferation [48-54] and soy isoflavones can bind to the estrogen receptor and either promote or inhibit estrogen receptor signaling depending on the level of endogenous estrogen [27,55]. Our data suggests that administration of a diet high in soy throughout embryonic and postnatal development can induce ER signaling in the mammary glands of pubertal mice. Genes with the greatest increase in expression in mammary tissue of soy-fed mice compared to the casein-fed mice were *Pgr* and *Areg*, both of which are regulated by ER signalling (Dragon Estrogen Responsive Genes Database [ERGDB] found at datam.i2r.a-star.edu.sg/ergdbV2/). *Areg* is a member of the epidermal growth factor family (EGFR) and is expressed at higher levels than any other EGF family member in the pubertal mammary gland [56]. The AREG protein mediates mammary epithelial proliferation in both ducts and terminal end buds [57,58]. Our data are the first report that dietary soy can influence *Areg* expression in mammary glands and thus provides a potential mechanism through which dietary soy can influence mammary epithelial proliferation and potentially transformation.

A number of other genes implicated in mammary gland development were evaluated in the mammary tissue of mice fed soy and casein diets including *Fgf10*, *Fgfr1*, *Fgfr2*, and *Tgfα* [59-61]. None of these genes were expressed at significantly different levels in the mammary glands of soy-fed mice compared to those of casein-fed mice suggesting that these genes are not contributing to soy’s influence on mammary tumor development.

One potential protective effect of soy observed in our study was a 3-fold reduction in the percentage of soy-fed mice with metastatic lesions compared to the casein-fed mice when the IGF-IR transgene was induced at 100 days of age. The difference in metastatic incidence however, was not significant due to the small number of casein-fed mice that developed mammary tumors. A decrease in the percentage of soy-fed mice with lung mestatases is consistent with most of the studies in the literature that demonstrate that soy or isoflavones inhibit mammary tumor migration and metastasis [62-66], however, at least one study found that isoflavones increased metastasis of MDA-MB-435 cells in immunocompromised mice [67]. It has been shown in breast, prostate and other cancers that soy isoflavones can inhibit cell detachment, invasion and the expression of proteases such as MMP-2 and MMP-9 [65,66,68]. The inhibitory effects of soy on tumor progression may explain the inverse relationship between soy consumption and breast cancer mortality and recurrence [69].

The consumption of high levels of dietary soy also appears to impact mammary tumor morphology. Mammary tumors from soy-fed mice more frequently contained a mixture of luminal and basal/mesenchymal cells than mammary tumors from casein fed mice. Tumors expressing luminal and basal cell types have been described in humans and classified as metaplastic breast cancers. This is a rare subtype of breast cancer representing 0.2-5% of all breast cancers and was the focus of a recent commentary in Nature Reviews Cancer [70]. Metaplastic breast cancers are typically ER-, PR- and HER2- and thus share features with triple negative breast cancer [70-73]. These tumors also typically express cytokeratins 5 and 14 and p63 [71,73,74], which is the staining pattern more frequently observed in mammary tumors from soy-fed mice than casein-fed mice in our study. Since this tumor subtype is rare little is known about it other than the fact that patients with metaplastic breast cancer typically have poor prognosis and do not respond well to conventional therapy [75]. There are no publications linking metaplastic breast cancer to soy diets or cultures that consume high amounts of dietary soy. However, soy consumption has been associated with metaplasia in other tissues. Squamous metaplasia and hyperplasia has been observed in the uterus of female rats fed genistein for 52 weeks [76,77]. Moreover, administration of genistein to a murine mammary or murine melanoma cell line induced the cells to acquire a spindle-shaped morphology [65]. As our understanding of metaplastic breast cancer improves, it will be interesting to see if dietary soy can influence the incidence of this breast cancer subtype.

## Conclusions

In conclusion, our study indicates that extremely high levels of dietary soy can promote mammary tumor development induced by overexpression of the tyrosine kinase receptor, IGF-IR. This increased susceptibility to transformation in the soy-fed mice appears to be associated with increased expression of *Areg*. Future studies should examine the impact of different levels of dietary soy on mammary tumor development and whether soy regulates *Areg* expression. Determining whether lower levels of dietary soy can inhibit mammary tumorigenesis and whether *Areg* expression has potential as a biomarker for predicting breast cancer risk may have important implications for prevention and treatment of this disease.

## Additional files

Additional file 1: Table S1. Nutritional information for the different diets utilized in this study. The casein diet represents the diet where the sole source of dietary protein was derived from casein while the soy diet represents the diet where the sole source of dietary protein was derived from isolated soy protein. The casein + DOXC and soy + DOXC represent the casein and soy diets containing 100 mg/kg of doxycycline.
Additional file 2: Figure S1. Mammary tumor onset and IGF-IR expression in response to different concentrations of doxycycline. Panel A shows the average mammary tumor onset as measured in days post IGF-IR transgene induction using concentration of doxycycline ranging from 100 mg/kg to 2000 mg/kg. Western blot analysis (B) and quantification of the blot (C) for IGF-IR protein induced by 2 g/kg DOXC or 100 mg/kg DOXC in MTB-IGFIR mice treated with DOXC for 14 days. The 2 g/kg DOXC induced significantly more IGF-IR protein than the 100 mg/kg DOXC, p < 0.05 and indicated by the asterisk. β-actin served as a loading control.
Additional file 3: Figure S2. Immunohistochemistry for IGF-IR in a mammary gland from casein-fed mice treated with DOXC that did not develop a mammary tumor (A) or a mouse that did not receive DOXC (B). IGF-IR protein was highly expressed (brown stain) in mammary epithelial cells of the DOXC-treated, casein-fed mice that did not develop tumors indicating that the IGF-IR transgene was still highly expressed in mammary tissue of these tumor-free, casein-fed mice. Scale bars, 100 μM.
Additional file 4: Figure S3. A box plot showing the level of staining for cytokeratin 5, cytokeratin 14 and p63 in mammary tumors from soy- and casein-fed mice. Tumors staining for each of the proteins in less than 1% of the tumor cells are indicated by light green boxes while tumors containing 1-5% positive cells are indicated by dark green boxes, 6-10% positive cells indicated by light red boxes and >10% positive cells indicated by dark red boxes.
Additional file 5: Figure S4. Immunohistochemistry for IGF-IR in a representative lung section showing that the lung metastases retain high levels of IGF-IR protein (A) that can be used to detect very small lung metastases (A,B). Scale bar in A is 100 μM while the scale bar in B is 33 μM.
Additional file 6: Figure S5. H&E stained section (A) and IGF-IR immunohistochemistry (B) of a lung from a casein-fed mouse that had extensive lung metastases in all lung lobes. H&E stained section of the primary mammary tumor that produced the extensive lung metastases is shown in (C). The histology of this tumor differed from most of the other tumors in that the mammary tumor cells still maintained glandular structures that were separated by stroma. Scale bars in A, B are 800 μM while the scale bar in C is 100 μM.
