# Supplementary material for: High levels of dietary soy decrease mammary tumor latency and increase incidence in MTB-IGFIR transgenic mice
Source: BMC Cancer. 2015 Feb 6;15:37. doi: 10.1186/s12885-015-1037-z (PMC4324669; doi:10.1186/s12885-015-1037-z)
Supplement: Additional file 1: Table S1. — Nutritional information for the different diets utilized in this study. The casein diet represents the diet where the sole source of dietary protein was derived from casein while the soy diet represents the diet where the sole source of dietary protein was derived from isolated soy protein. The casein + DOXC and soy + DOXC represent the casein and soy diets containing 100 mg/kg of doxycycline. [file 12885_2015_1037_MOESM1_ESM.docx]

| Nutrient | Casein Diet (g/kg) | Soy Diet (g/kg) | Casein + DOXC (g/kg) | Soy + DOXC (g/kg) |
| --- | --- | --- | --- | --- |
| Casein | 200 | 0 | 200 | 0 |
| Isolated Soy Protein* | 0 | 200 | 0 | 200 |
| Doxycycline Hyclate | 0 | 0 | 0.115 | 0.115 |
| L-Cystine | 4.3 | 2.8 | 4.3 | 2.8 |
| L-Methionine | 0 | 2.8 | 0 | 2.8 |
| Corn Starch | 390.3 | 393.8 | 390.2 | 393.7 |
| Maltodextrin | 132 | 132 | 132 | 132 |
| Sucrose | 100 | 100 | 100 | 100 |
| Soybean Oil | 70 | 63 | 70 | 63 |
| Cellulose | 50 | 50 | 50 | 50 |
| Mineral Mix, AIN-93G-MX | 35 | 35 | 35 | 35 |
| Calcium Phosphate, dibasic | 3.1 | 5.3 | 3.1 | 5.3 |
| Calcium Carbonate | 1.0 | 1.0 | 1.0 | 1.0 |
| Magnesium Oxide | 0.154 | 0.154 | 0.154 | 0.154 |
| Cupric Carbonate | 0.0038 | 0.0038 | 0.0038 | 0.0038 |
| Ferric Citrate | 0.2352 | 0.2352 | 0.2352 | 0.2352 |
| Sodium Selenite | 1.25 | 1.25 | 1.25 | 1.25 |
| Vitamin Mix, AIN-93-VX | 10 | 10 | 10 | 10 |
| Choline Bitartrate | 2.5 | 2.5 | 2.5 | 2.5 |
| Vitamin K_1_, phylloquinone | 0.0003 | 0.0003 | 0.0003 | 0.0003 |
| Vitamin B_12_ | 0.025 | 0.025 | 0.025 | 0.25 |
| TBHQ, antioxidant | 0.014 | 0.014 | 0.014 | 0.014 |
| Red Food Color | 0.15 | 0 | 0 | 0 |
| Pink Food Color | 0 | 0.15 | 0 | 0 |
| Yellow Food Color | 0 | 0 | 0.15 | 0 |
| Blue Food Color | 0 | 0 | 0 | 0.15 |

Table S1. Dietary Components of the Different Diets

*Isolated soy protein contains ~463, 95, 933, 101, 57, and 9 ppm of daidzin, daidzein, genistin, genistein, glycetin, and glycetein, respectively with a total of ~1660 ppm, all aglycone
