# Supplementary figures and images for: High levels of dietary soy decrease mammary tumor latency and increase incidence in MTB-IGFIR transgenic mice
Source: BMC Cancer. 2015 Feb 6;15:37. doi: 10.1186/s12885-015-1037-z (PMC4324669; doi:10.1186/s12885-015-1037-z)

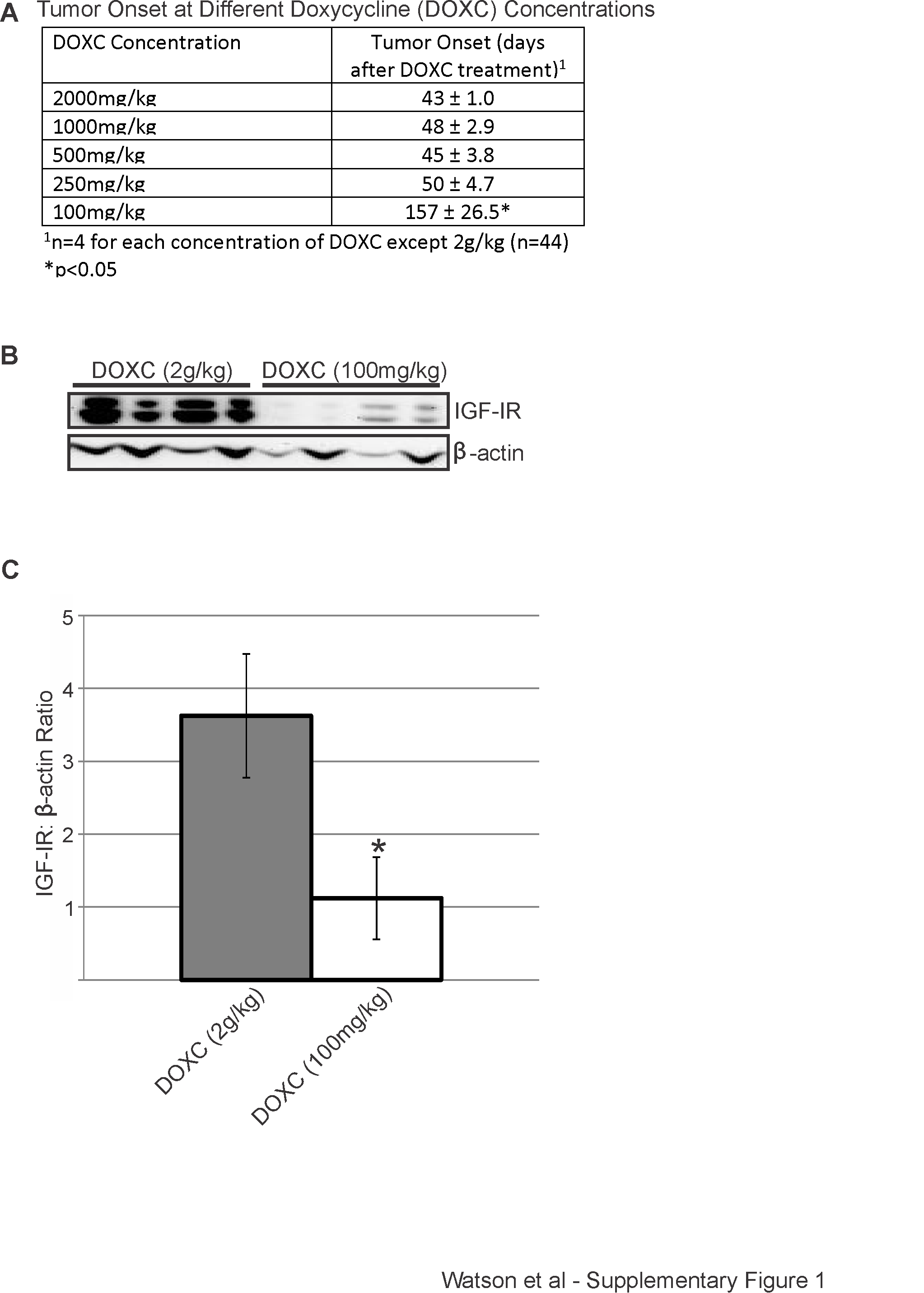

Supplement: Additional file 2: Figure S1. — Mammary tumor onset and IGF-IR expression in response to different concentrations of doxycycline. Panel A shows the average mammary tumor onset as measured in days post IGF-IR transgene induction using concentration of doxycycline ranging from 100 mg/kg to 2000 mg/kg. Western blot analysis (B) and quantification of the blot (C) for IGF-IR protein induced by 2 g/kg DOXC or 100 mg/kg DOXC in MTB-IGFIR mice treated with DOXC for 14 days. The 2 g/kg DOXC induced significantly more IGF-IR protein than the 100 mg/kg DOXC, p < 0.05 and indicated by the asterisk. β-actin served as a loading control. [file 12885_2015_1037_MOESM2_ESM.tiff]

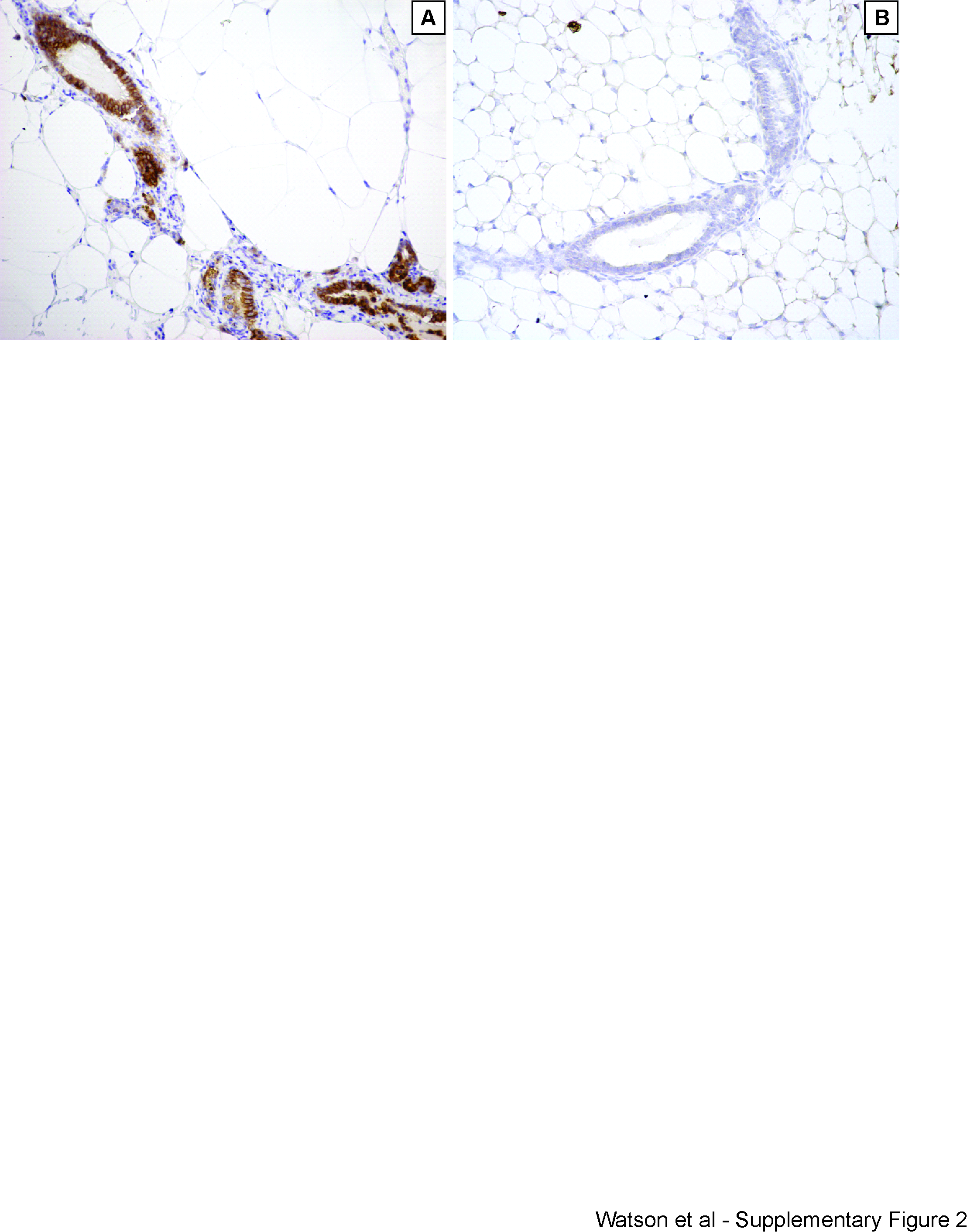

Supplement: Additional file 3: Figure S2. — Immunohistochemistry for IGF-IR in a mammary gland from casein-fed mice treated with DOXC that did not develop a mammary tumor (A) or a mouse that did not receive DOXC (B). IGF-IR protein was highly expressed (brown stain) in mammary epithelial cells of the DOXC-treated, casein-fed mice that did not develop tumors indicating that the IGF-IR transgene was still highly expressed in mammary tissue of these tumor-free, casein-fed mice. Scale bars, 100 μM. [file 12885_2015_1037_MOESM3_ESM.tiff]

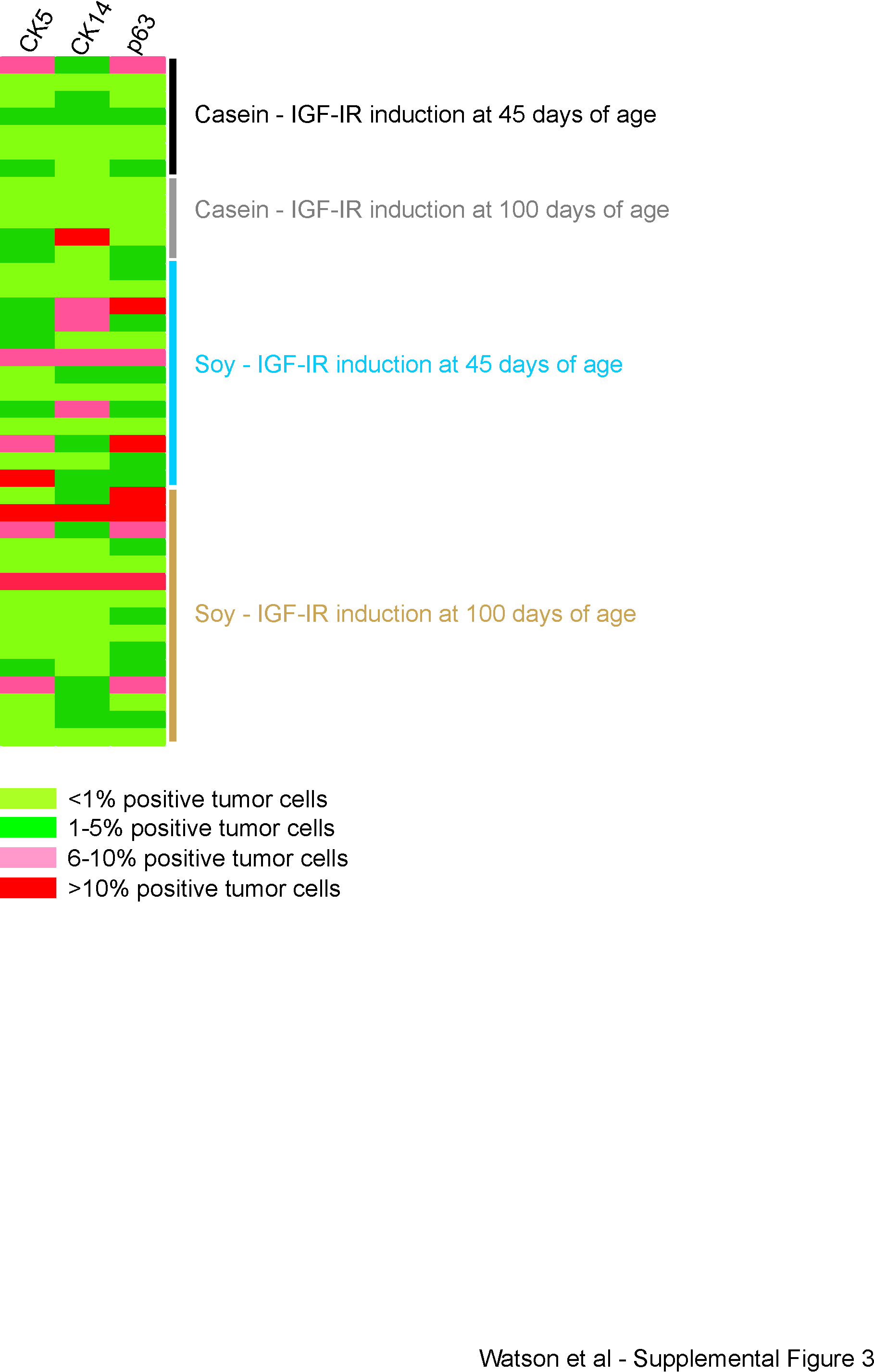

Supplement: Additional file 4: Figure S3. — A box plot showing the level of staining for cytokeratin 5, cytokeratin 14 and p63 in mammary tumors from soy- and casein-fed mice. Tumors staining for each of the proteins in less than 1% of the tumor cells are indicated by light green boxes while tumors containing 1-5% positive cells are indicated by dark green boxes, 6-10% positive cells indicated by light red boxes and >10% positive cells indicated by dark red boxes. [file 12885_2015_1037_MOESM4_ESM.tiff]

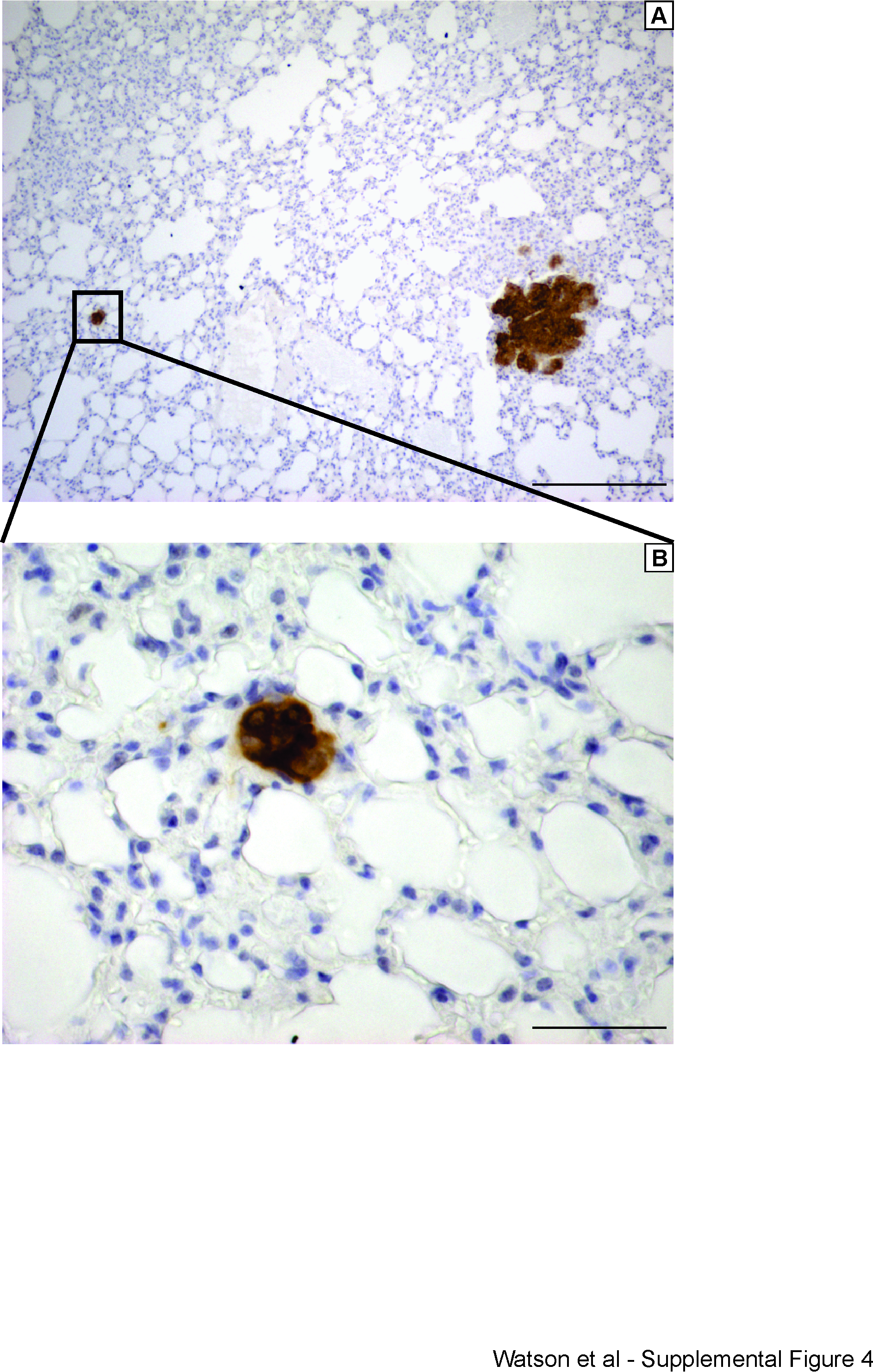

Supplement: Additional file 5: Figure S4. — Immunohistochemistry for IGF-IR in a representative lung section showing that the lung metastases retain high levels of IGF-IR protein (A) that can be used to detect very small lung metastases (A,B). Scale bar in A is 100 μM while the scale bar in B is 33 μM. [file 12885_2015_1037_MOESM5_ESM.tiff]

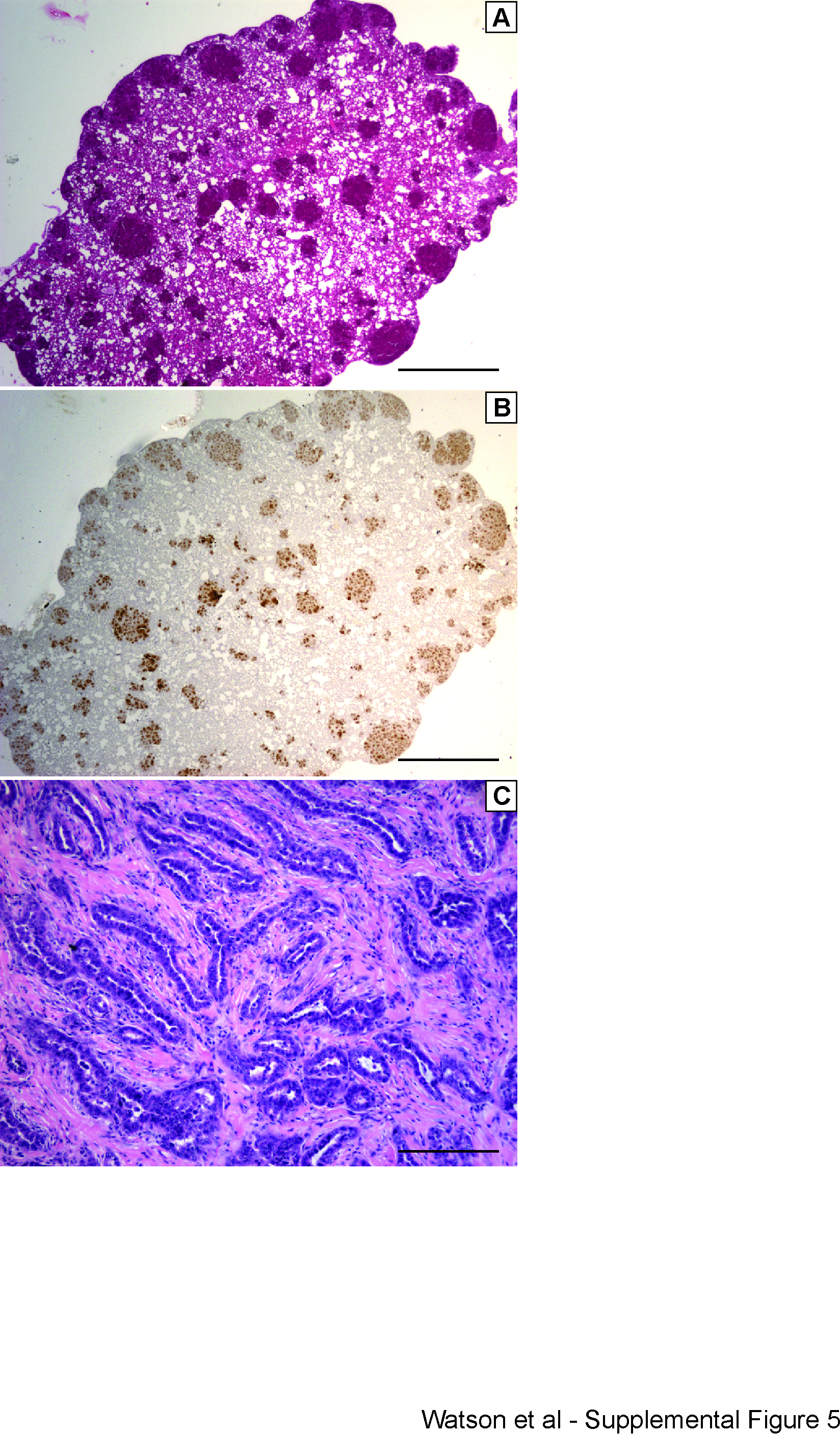

Supplement: Additional file 6: Figure S5. — H&E stained section (A) and IGF-IR immunohistochemistry (B) of a lung from a casein-fed mouse that had extensive lung metastases in all lung lobes. H&E stained section of the primary mammary tumor that produced the extensive lung metastases is shown in (C). The histology of this tumor differed from most of the other tumors in that the mammary tumor cells still maintained glandular structures that were separated by stroma. Scale bars in A, B are 800 μM while the scale bar in C is 100 μM. [file 12885_2015_1037_MOESM6_ESM.tiff]
